# Emergence of *Brucella suis* in dogs in New South Wales, Australia: clinical findings and implications for zoonotic transmission

**DOI:** 10.1186/s12917-016-0835-0

**Published:** 2016-09-09

**Authors:** Siobhan M. Mor, Anke K. Wiethoelter, Amanda Lee, Barbara Moloney, Daniel R. James, Richard Malik

**Affiliations:** 1 Faculty of Veterinary Science, The University of Sydney, Sydney, 2006 NSW Australia; 2 Tufts University School of Medicine, 145 Harrison Avenue, Boston, 02111 MA USA; 3 New South Wales Department of Primary Industries, Woodbridge Road, Menangle, 2568 NSW Australia; 4 New South Wales Department of Primary Industries, 161 Kite Street, Orange, 2800 NSW Australia; 5 Small Animal Specialist Hospital, 1 Richardson Place, North Ryde, 2113 NSW Australia

**Keywords:** Brucellosis, *Brucella suis*, Dog, Emergence, Zoonosis, Australia

## Abstract

**Background:**

Animal reservoirs of brucellosis constitute an ongoing threat to human health globally, with foodborne, occupational and recreational exposures creating opportunities for transmission. In Australia and the United States, hunting of feral pigs has been identified as the principal risk factor for human brucellosis due to *Brucella suis*. Following increased reports of canine *B. suis* infection, we undertook a review of case notification data and veterinary records to address knowledge gaps about transmission, clinical presentation, and zoonotic risks arising from infected dogs.

**Results:**

Between 2011 and 2015, there was a 17-fold increase in the number of cases identified (74 in total) in New South Wales, Australia. Spatial distribution of cases largely overlapped with high feral pig densities in the north of the state. Ninety per cent of dogs had participated directly in pig hunting; feeding of raw feral pig meat and cohabitation with cases in the same household were other putative modes of transmission. Dogs with confirmed brucellosis presented with reproductive tract signs (33 %), back pain (13 %) or lameness (10 %); sub-clinical infection was also common (40 %). Opportunities for dog-to-human transmission in household and occupational environments were identified, highlighting potential public health risks associated with canine *B. suis* infection.

**Conclusions:**

Brucellosis due to *B. suis* is an emerging disease of dogs in Australia. Veterinarians should consider this diagnosis in any dog that presents with reproductive tract signs, back pain or lameness, particularly if the dog has a history of feral pig exposure. Moreover, all people in close contact with these dogs such as hunters, household contacts and veterinary personnel should take precautions to prevent zoonotic transmission.

## Background

In a recent global review of diseases at the wildlife-livestock interface, brucellosis ranked amongst the top ten diseases [1]. Because the disease is readily transmitted between wildlife, domestic animals and humans, new and re-emerging foci represent an ongoing challenge worldwide with foodborne and occupational exposures to livestock and livestock products recognised as the main traditional risk factors in humans [2]. Increasingly, recreational activities such as hunting of feral animals and wildlife have emerged as an alternative risk factor [3]. Out of the four terrestrial zoonotic *Brucella* species – *B. melitensis*, *B. abortus*, *B. suis*, and *B. canis* – only *B. abortus* and *B. suis* have been frequently found in wildlife [4]. In particular, contact with bison (*Bison bison*), elk (*Cervus elaphus*) or African buffalo (*Syncerus caffer*) as well as reindeer (*Rangifer tarandus*) have been identified as important risk factors for human brucellosis due to *B. abortus* [3, 5, 6] and *B. suis* biovar 4 [7], respectively.

Australia is currently free of many of the human pathogenic *Brucella* species; *B. melitensis* and *B. canis* are exotic and *B. abortus* was eradicated from cattle and buffalo by 1989 [8]. However, *B. suis* biovar 1 is endemic in feral pigs (*Sus scrofa*), and was thought to be limited to east Queensland (QLD) [9–12] until recently when seropositive feral pigs were identified in northern New South Wales (NSW) [13]. Hunting and dressing of carcasses of feral pigs has been associated with human *B. suis* biovar 1 infections in Australia [14, 15] and the United States (US) [16].

Feral pigs are one of the most successful invasive species worldwide due to their adaptable, highly reproductive and opportunistic omnivore nature [17]. Due to the severely negative impacts on crop and livestock farming as well as wildlife predation and habitat degradation, they are regarded as a threat to biodiversity in Australia. Consequently, land owners in the state of NSW are required by law to institute control measures (e.g. hunting, trapping or poisoning) on their properties [18]. Hunting is also permitted on public land and state forests [19]. An estimated 100–200,000 hunters kill up to 500,000 feral pigs per year in Australia [20] and dogs are widely used to bail, locate and hold feral pigs [21].

Since 2011, a growing number of *B. suis* infections have been reported in dogs in NSW. Prior to 2011, the only published evidence for canine infection in Australia was a single laboratory report citing isolation of *B. suis* from a canine testis in QLD in 1968 [12]. However, sporadic case reports from other countries confirm that dogs can be infected [22–33]. Since *B. suis* biovar 1 is second only to *B. melitensis* in terms of pathogenicity for humans [34], concerns about the potential for dog-to-human transmission in NSW initially led to the recommendation that affected dogs be euthanized [35]. However, knowledge of the natural history of infection, clinical presentation and zoonotic implications of canine *B. suis* infection is meagre and the policy is under review. There is a need for better scientific evidence to underpin sound, risk-based policy responses.

To address these gaps, we documented the epidemiology and clinical findings of canine *B. suis* cases diagnosed between 2011 and 2015. Whether infected dogs pose an ongoing threat to their owners and household contacts and/or other dogs is of principal interest to policy makers. Thus, we examined exposure histories of affected dogs with a view to expanding current understanding of modes of acquisition in dogs. We also reviewed veterinary records to identify opportunities for occupational and household exposure.

## Methods

Data on cases notified between 1 January 2011 and 31 December 2015 were obtained from NSW Department of Primary Industries (DPI), which incorporates the State Veterinary Diagnostic Laboratory (SVDL). SVDL is the only veterinary laboratory that performs serological testing for brucellosis in NSW. As a notifiable disease, other laboratories which diagnose cases through other means (e.g. culture) are required to report cases to DPI. To ensure that no cases had been unreported, DPI staff contacted private laboratories to request information on cases they had diagnosed.

Suspect cases were initially screened at SVDL using the sensitive Rose-Bengal agglutination test (RBT). Dogs with RBT agglutination scores of 1+ (low positive) to 3+ (high positive) were subjected to confirmation using the more specific complement fixation test (CFT). A positive case was defined as a dog with positive culture, or positive RBT and reciprocal CFT titre ≥16. Since neither of these serological tests is perfect, dogs with positive RBT, but anti-complimentary CFT or reciprocal CFT titre <16 and history (pig hunting, eating raw feral pig meat, contact with a positive case) and/or indicative clinical signs were considered inconclusive cases. Data accompanying the laboratory records included: date of blood collection, name and location of referring veterinarian, serology results, signalment (gender, breed, age), and history as provided in the laboratory submission form or obtained during follow-up of cases by DPI. In addition, data on the total number of dogs tested were obtained from DPI. Dogs that underwent repeat testing were only counted once. Following preliminary analysis, veterinarians attending cases were contacted and invited to share the full records of affected dogs. Owner details were removed from records to ensure confidentiality. A unique identifier was retained enabling dogs with the same owner to be linked.

To assess temporal trends, the number of dogs tested and positive/inconclusive cases identified annually were plotted in R (v3.1.3, R Foundation, Vienna, Austria) taking into consideration repeat testing of individual animals. Spatial distribution of cases aggregated by town was mapped in ArcMAP (v10; ESRI, Redlands, CA). Data on feral pig density was obtained from DPI surveys conducted in 2009 [36]. To examine transmission pathways at household level, network analysis was performed using the igraph package in R [37]. A cluster was defined as a household that included ≥1 dog that tested positive/inconclusive for *B. suis*.

Demographic and clinical information were extracted from veterinary records, tabulated in Excel and summarized as counts and percentages. Fisher’s exact test was used to investigate associations between clinical presentation and signalment. As body temperature was not reliably documented in the majority of cases, we elected not to report on this measurement. We note, however, that fever was not invariably present in dogs for which temperature was recorded. Where absence of other clinical signs was not specifically mentioned in the case notes, these were presumed not to be present. Sub-clinical cases were defined as cases that did not have any clinical signs consistent with brucellosis but which were reactive on RBT/CFT.

## Results

Between 2011 and 2015, 437 unique dogs were tested for brucellosis at SVDL, of which 72 (16.5 %) were seroreactive (46 positive, 26 inconclusive cases). One additional case was notified to DPI but was excluded since the dog resided in QLD. A further case was excluded because it was identified as part of a research study and the dog was not subjected to evaluation sufficient to determine exposure history or clinical status. During follow-up with private laboratories, a further two cases (both positive) were identified. Thus, we present the clinical findings from 74 dogs, diagnosed by either SVDL or private laboratories. Veterinary records and/or additional information were provided by the referring veterinarian for 50 of these 74 cases (67.6 %). Since information on possible in-contact animals was not available for the cases diagnosed by private laboratories, assessment of household clustering and potential exposure pathways was limited to the 72 cases diagnosed at SVDL.

### Epidemiology and transmission

**Fig. 1:**
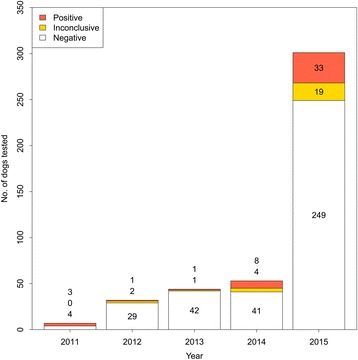
Number of dogs tested for *Brucella suis* in New South Wales, Australia (*n* = 497)

**Fig. 2:**
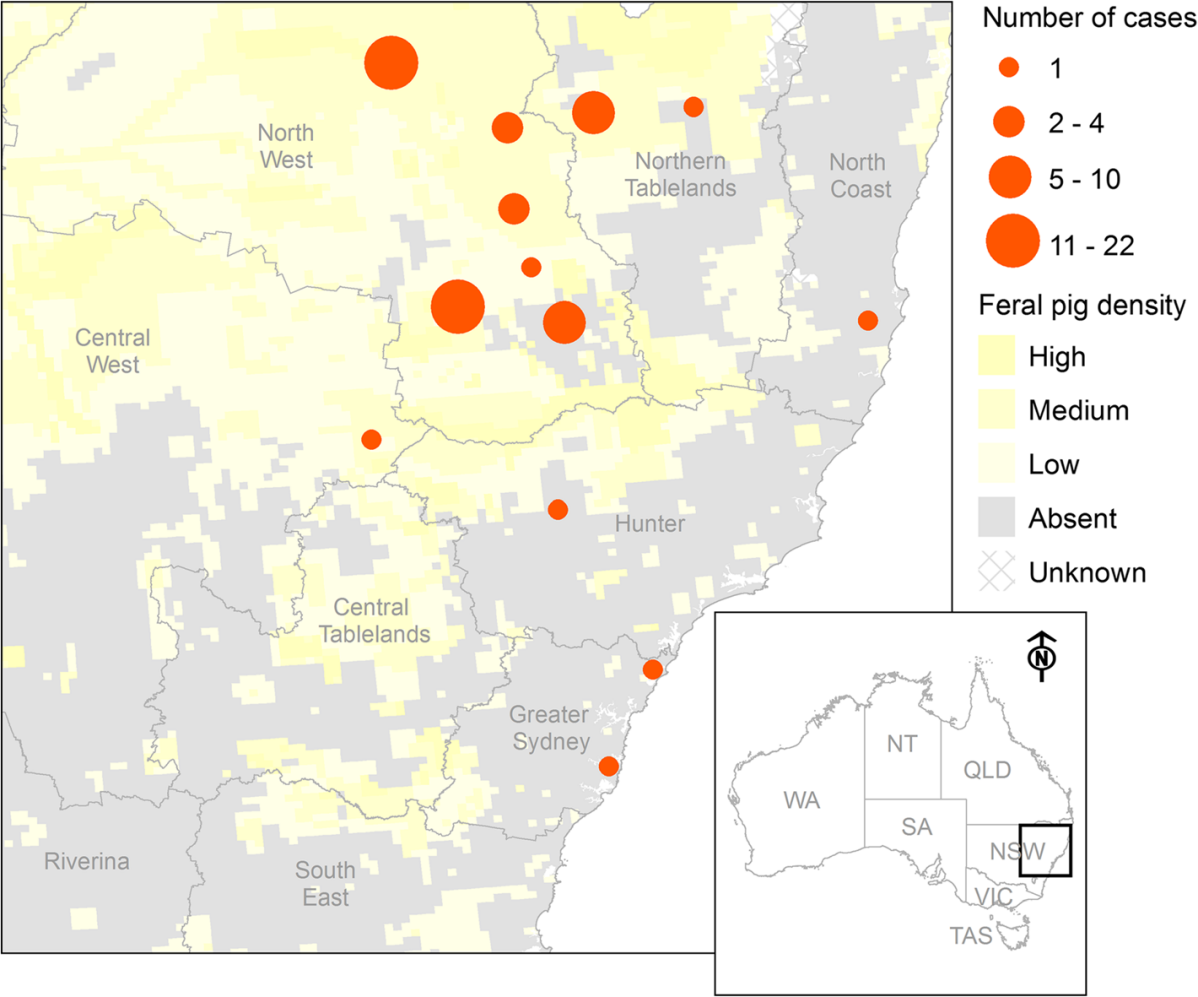
Spatial distribution of dogs tested positive/inconclusive for *Brucella suis*. Data from 2011 to 2015 (*n* = 74) is aggregated to the level of the town in which the referring veterinary practice is located. For contrast, feral pig density (estimated in 2009) is also shown

Figures 1 and 2 show the temporal trend and geographic distribution of cases, respectively. The proportion of positive/inconclusive dogs increased from around 9 % in 2012/13 to 17–22 % in 2014/15. Cases were largely spatialised to northern NSW, where feral pig density is highest.

**Fig. 3:**
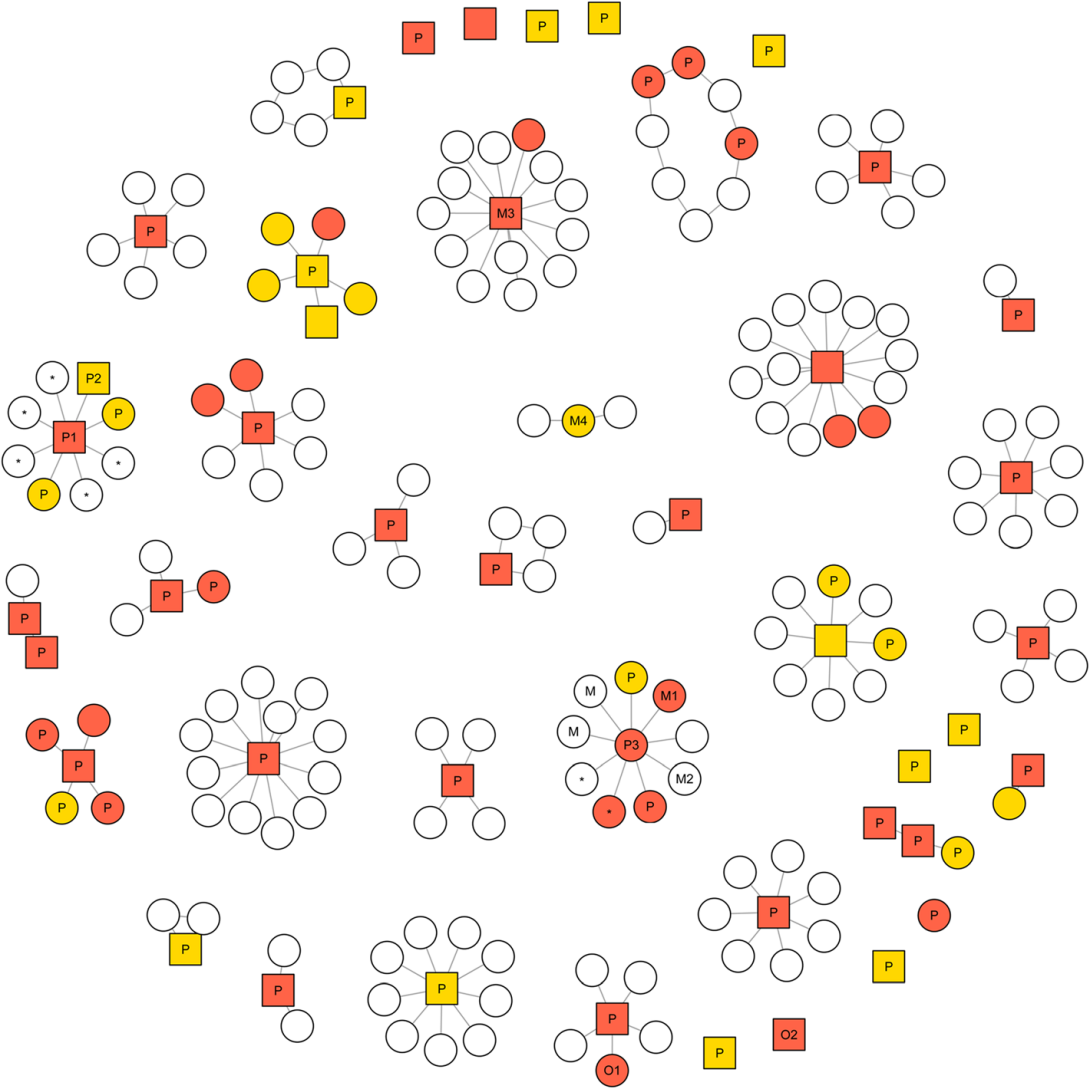
Network analysis of canine *Brucella suis* cases in New South Wales, Australia. Positive (*red*), inconclusive (*orange*) and negative (*white*) dogs clustered within the same household. Index cases and in-contact dogs appear as hub and spoke, while dogs presenting simultaneously appear as other formations. Dogs with and without clinical signs are depicted as squares and circles, respectively. Relevant exposure histories are shown where known (P = pig hunting, M = fed raw feral pig meat, * = offspring of seropositive dog(s), O = other exposure history). See main text for further description of individual animals (P1, P2, P3, M1, M2, M3, M4, O1 and O2)

Forty one discrete clusters were reported to SVDL (Fig. 3). Records revealed linkages across clusters. For example, dogs from different households were sometimes taken on the same hunting trips. Typically, index cases presented with clinical findings consistent with brucellosis or following injuries sustained during pig hunting. Clinical suspicion led to testing of these index cases, confirmation of which led to further testing of in-contact animals and detection of sub-clinical cases in the same household. Only three index cases presented sub-clinically; in all cases there was a history of pig hunting and/or feeding of raw feral pig meat. The two earliest clusters in NSW had links to QLD. The first (October 2011) involved a dog that had been pig hunting in QLD. The second (June 2012) involved a dog mated to a female ‘pig dog’ from QLD 5 weeks earlier. The latter dog was used for pig hunting but had not been hunting in QLD for 4 years.

Potential exposure histories were available for 57/72 cases diagnosed by SVDL (38/41 household clusters). Pig hunting was practiced by 36/38 households and was the most plausible source of infection in 51/57 cases (89.4 %). However, within pig hunting households, not all seroreactive dogs had participated in this activity (Fig. 3: M1, fed raw feral pig meat; O1, no known contact with feral pigs).

Serology was performed on offspring of seroreactive dogs in two pig hunting households. In one household the female (Fig. 3: P1) and male (P2) canine parents tested seropositive and inconclusive, respectively, while the 5-month old offspring tested seronegative. Review of veterinary records revealed that 2 pups from the same litter had been aborted prematurely; the remaining five pups were delivered by caesarean. In the other household, the female (P3) tested seropositive when her offspring were 12 months old; only one of the two offspring (full siblings) was seropositive on subsequent testing. Another dog in this household (M2) was inseminated by a dog that later tested positive (M1), although the former was found to be seronegative on subsequent testing.

Pig hunting status was not known for the three remaining household clusters. In two of these clusters, index cases had a history of being fed raw feral pig meat (Fig. 3: M3, M4) and were the only seropositive cases detected in these households. The remaining dog (O2) had no known contact with feral pigs or infected dogs and was not fed raw feral pig meat according to the current owner, although this dog was adopted from a pound and exposure history prior to this was unobtainable.

### Clinical presentation and outcome in dogs

Tables 1 and 2 show the signalment and clinical presentation of dogs, respectively. The median age of affected dogs was 2 years (range 5 months to 12 years). There were no clear relationships between age and clinical presentation, with dogs aged ≤24 months just as likely to present with certain clinical signs as those aged >24 months (*p* > 0.05 for all signs). De-sexed dogs were more likely than reproductively intact animals to present with back pain (50 % versus 10.3 %; *p* = 0.074) and lameness (50 % versus 7.4 %, *p* = 0.045), while females were more likely than males to be sub-clinically affected (53.6 % versus 31.8 %, *p* = 0.087). No other significant associations existed between gender, reproductive status and specific signs. Dogs with clinical signs were just as likely as dogs without clinical signs to have an inconclusive sero-status (35 % versus 37.5 %, *p* = 1.0).

**Table 1:** Signalment of dogs diagnosed with *Brucella suis* in New South Wales, Australia (*n* = 74)

Characteristic	Positive (*n* = 48) No. (%)	Inconclusive (*n* = 26) No. (%)
Age		
≤24 months	16 (33)	6 (23)
>24 months	26 (54)	18 (69)
Unknown	6 (13)	2 (8)
Gender		
Male intact	26 (54)	16 (62)
Male de-sexed	1 (2)	1 (4)
Female intact	17 (35)	9 (35)
Female de-sexed	2 (4)	0
Unknown	2 (4)	0
Breed		
Boxer/Boxer X^a^	5 (10)	0
Bull Arab/Bull Arab X	11 (23)	5 (19)
Bull Mastiff/Bull Mastiff X	9 (19)	6 (23)
Cattle dog/Collie/Kelpie	5 (10)	3 (12)
Wolfhound/Wolfhound X	2 (4)	4 (15)
Pig dog, unspecified breed	6 (13)	5 (19)
Other^b^	8 (17)	3 (12)
Unknown	2 (4)	0

Cases from 2011 to 2015 (*n* = 74) are presented

^a^X - cross-breed

^b^Other includes: Great Dane/Great Dane X (*n* = 3), Bull Terrier/Bull Terrier X (2), Catahoula (1), Dachshund (1), Jack Russell Terrier (1), Staffordshire Terrier (1), Staghound (1), and cross-bred, breeds unspecified (1)

**Table 2:** Clinical presentation of dogs diagnosed with *Brucella suis* in New South Wales, Australia (*n* = 74)

Clinical presentation	Positive (*n* = 48) No. (%)	Inconclusive (*n* = 26) No. (%)
Sub-clinical infection^a^	19 (40)	12 (46)
Male	8 (30)	6 (35)
Female	9 (47)	6 (67)
Reproductive tract signs^b^	14 (33)	11 (44)
Orchitis/epididymitis^c^	12 (46)	9 (56)
History of abortion^c^	2 (12)	2 (22)
Lethargy/‘off-colour’	13 (27)	8 (31)
Discospondylitis/ back pain	6 (13)	3 (12)
Intact	4 (9)	3 (12)
De-sexed	2 (67)	0
Lameness	5 (10)	2 (8)
Intact	3 (7)	2 (8)
De-sexed	2 (67)	0
Other^d^	7 (15)	4 (15)

Cases from 2011 to 2015 (*n* = 74) are presented. Denominators are adjusted for gender and reproductive status, where applicable

^a^Gender of two sub-clinically affected dogs was unknown (see Table 1)

^b^Reproductively intact animals only (see Table 1)

^c^Other includes: superficial abscess (*n* = 4), haematuria (2), prostatitis (2), vomiting (2), weight loss (2), lymphoadenomegaly (2), suppurative endometritis (1)

Signs consistent with reproductive tract involvement were the most common presenting problem and occurred across a range of ages (1–9 years). Among 21 dogs with orchitis/epididymitis, presentation was unilateral in 12 cases and bilateral in eight (one unspecified). Serology conducted on one female at the time of abortion was negative; retesting 8 months later however showed an inconclusive result.

Nine dogs presented with back pain, four of which also presented with intermittent lameness, while one presented with concurrent reproductive tract signs. Back pain was mostly localized to the thoracolumbar junction (five out of six cases). Two dogs were diagnosed with discospondylitis with localized empyema adjacent to affected vertebrae. While there was no bacterial growth following culture of cerebrospinal fluid, *B. suis* was cultured from soft tissue material collected during decompressive hemilaminectomy in both animals.

Three dogs presented with lameness without back pain. In one case (Bull-Mastiff, 22 months), the dog presented with a history of shifting lameness and swollen joints followed by an acute episode of dyspnoea. A large, oedematous mass cranial to the larynx was palpable and considered to be impeding airflow. Generalised lymphadenomegaly was also evident and unilateral orchitis/epididymitis and pyrexia developed one week later. The dog was castrated and euthanised following culture of *B. suis* biovar 1 from the affected testis. A second dog (Kelpie X, 33 months) presented with painful, dorsally-swollen left carpus. *B. suis* biovar 1 was cultured from joint fluid collected from the inter-carpal joint.

### Public health considerations

In two separate clusters, veterinary records indicated that the dogs’ owner had been diagnosed with brucellosis prior to presentation of the dog. Both households practiced pig hunting. Other potentially risky practices identified from veterinary records included assistance with whelping and on-sale of live-born offspring prior to diagnosis in the parent dogs. A woman in at least two households was reported as being pregnant at the time of canine diagnosis.

Opportunities for potential occupational exposure were noted in records of several cases. Four dogs were de-sexed during the episode of illness as part of clinical management of reproductive tract signs, while two underwent spinal surgery. Infection status of these dogs was unknown at the time of the procedure, and thus the surgical teams are presumed to have taken no special precautionary measures to minimise the risk of infection. Likewise, bacterial culture was performed by unsuspecting laboratory staff involved in the diagnosis of two cases.

## Discussion

This article comprises the largest and most detailed compilation of canine *B. suis* cases reported to date. Previous cases have been recorded in Bulgaria [31], Brazil [25, 28, 29], Germany [27], Hungary [26], India [23] and the US [22, 24, 30, 32]. Many of these were documented in the mid-20^th^ century when commercial piggeries constituted the main reservoir of *B. suis* biovar 1. With eradication having been achieved in many of these countries, infection dynamics have shifted considerably, as have exposure risks [16].

We observed a 17-fold increase in the number of canine cases detected in NSW between 2011 and 2015. A similar trend has been documented in Georgia, US in association with recreational pig hunting activities [22]. The extent to which this increase reflects true emergence versus enhanced detection of previously unrecognized foci of infection is unclear. Certainly awareness for the disease is growing in NSW, as evidenced by the increasing number of dogs presented for testing. However, the incursion of seropositive feral pigs in NSW lends biological plausibility to a novel source of infection, a finding supported by the high degree of spatial overlap between feral pigs and canine cases reported here. We suspect *B. suis* was introduced following deliberate transportation of feral pigs across state borders by recreational hunters, a practice which is illegal but, anecdotally, widely practiced [20]. Natural migration of infected feral pigs across the NSW-QLD border is also likely [13]. Movements of up to 12 km per day as well as swimming across rivers and off coastlines have been observed in feral pigs and seem to be largely driven by external factors such as human disturbance, weather conditions, food and habitat availability [38].

Given co-occurrence of exposures in the same households and potential for casual contact between dogs (e.g. through sharing feed bowls), it is difficult to draw definitive conclusions about transmission. Direct involvement in pig hunting was the most plausible mode of transmission for the majority (90 %) of dogs, while feeding of raw feral pig meat resulted in infection of dogs not involved directly with hunting. Evidence for vertical transmission was limited in cases reported here, although we presume it occurs by extrapolation from other *Brucella* spp. [39]. Pig-to-dog transmission of *B. suis* through hunting or co-habitation with domestic pigs has been reported [22, 28]. Precisely how dogs acquire the infection from pigs is not known. Given the nature of pig hunting – which involves a high frequency of injuries to both animals and humans – we suspect transfer likely occurs through blood-borne contact and/or direct inoculation by contamination of wounds, transmission via mucous membranes or via ingestion of pig offal or meat. Ingestion of aborted foetal material has also been proposed [27]. A number of dogs in this study presented for treatment of sub-cutaneous abscesses most likely sustained as a result of pig hunting. Cutaneous lesions due to *B. suis* have been described in humans [40] and may be consistent with traumatic inoculation at these sites.

Little is published on the risk of foodborne transmission of *B. suis* biovar 1 via meat, although the first canine case was linked to this practice [33]. Further, Hellmann and Sprenger [27] postulated that raw pet food obtained from Eastern European countries was the likely source of infection for dogs in Germany. The likelihood that *B. suis* may be transmitted via feral pig meat has implications for human and animal health. At one time Australia supplied as much as 30 % of wild boar meat consumed globally, with a smaller quantity being sold domestically and by-products/substandard carcasses used as pet food [41]. Proper cooking and/or canning will destroy *Brucella* spp., thus the main risk comes from handling and consumption of undercooked meat [42]. Raw pet food diets are becoming popular in Australia and elsewhere, and concerns have been raised following detection of a number of zoonotic foodborne hazards in raw pet food [43, 44]. We are not aware of any studies that have specifically tested commercial raw food diets for *B. suis* in Australia or elsewhere.

It is generally assumed that hunters acquire brucellosis following direct contact with blood and other fluids and/or aerosols during field slaughter of feral pigs [14, 42]. Risk reduction strategies have therefore focused on preventing transmission at slaughter, including promoting use of personal protective clothing, such as mesh gloves to prevent abrasions and cuts to the hands [45]. The extent to which infected dogs present an ongoing risk to humans in the household is unknown but plausible in the view of the authors. A single 1967 case report found that a woman in Massachusetts most likely acquired *B. suis* following unprotected disposal of aborted dog foetuses [30]. The dog, which was confirmed at necropsy to be infected, had been allowed to roam freely and likely acquired the infection from a swine farm nearby. Other case reports have implicated dogs as the source of human infection with *B. abortus* [46], *B. canis* [47, 48], and *B. melitensis* [49, 50], establishing that pathogen excretion and/or contact sufficient to lead to human infection can occur.

In reviewing veterinary records, opportunities for human exposure through contact with body fluids within a household environment (e.g. aborted foetuses, placenta) as well as occupational exposure of veterinary staff via routine (e.g. diagnostic specimen collection and processing, castration and ovariohysterectomy) and advanced procedures (e.g. spinal surgery) were identified. High speed burr was used in the latter case, increasing risk of aerosolisation. Veterinary staff should be encouraged to adopt strict precautions, including use of masks, gloves and eye shields when handling dogs with a history of pig hunting. Further, following presumptive diagnosis by serology, preliminary antimicrobial therapy may make subsequent surgery less hazardous by reducing viability of organisms in vivo. We also recommend that specimens collected from pig hunting dogs be clearly identified as such, so that laboratory staff can perform subsequent manipulations under safe conditions even if specimens are not specifically tested for brucellosis. Finally, we advise that pregnant women (in the household and in the workplace) should avoid contact with hunting dogs.

Dogs with brucellosis presented with one of three syndromes consistent with involvement of the reproductive tract, axial skeleton or appendicular skeleton, although combinations were possible also. Reproductive tract involvement is recognized as a clinical feature of brucellosis in a number of animal species, including dogs [25, 27, 29, 33]. Discospondylitis due to *B. suis* has also been previously described in one dog [24] and spondylitis is a well-known complication of *B. suis* infections in humans [51]. The detailed clinical findings and successful treatment of one discospondylitis case reported here (Fig. 3: O2) as well as two orchitis cases is described elsewhere (James et al. 2016, *in review*). We found that dogs that had been de-sexed were more likely to present with back pain and/or lameness than reproductively intact animals, suggesting that *B. suis* may display tropism for bones or joints when the preferred site (reproductive tract) is not present.

A considerable number of dogs without clinical signs were reactive on RBT/CFT and were presumed to be sub-clinically infected. These cases present a particular challenge in terms of detection and clinical management. Sub-clinical infection has been reported in both naturally occurring [26] and experimental infections in dogs [52]. The finding that females were more likely to be sub-clinically infected may reflect a bias towards detection in males, with owners more likely to recognize and investigate enlarged testes. Alternatively, veterinary records may have failed to adequately document a history of abortion in healthy female dogs that present later for testing. Given the high frequency of sub-clinical infection, regular testing of pig hunting dogs and in-contact animals, particularly prior to invasive surgeries or breeding, is advised.

This study has a number of limitations. Clinical and exposure histories were limited for some dogs, making conclusions about source of infection and onset of illness difficult in these cases. Further, the location and timing of hunting trips was not disclosed. Such information would be useful in guiding surveillance and control activities in the feral pig population, as well as updating knowledge on incubation period in dogs. Diagnosis remains a particular challenge with a number of dogs deemed to have inconclusive serological results. To our knowledge, necropsy was not performed on any dog after euthanasia nor was sequential serology performed on animals that were not euthanised (with exception of the case described by James et al., *in review*). Culture is the gold standard for diagnosing brucellosis but was not pursued in the majority of cases. The sensitivity and specificity of the serological tests used has not been determined, nor is it known whether seropositive cases are actively infected or rather previously exposed but with elimination of all foci of disease. This distinction has major implications for evaluating public health risks associated with canine infection and, for this reason, molecular-based methods are being pursued by DPI.

## Conclusions

In conclusion, brucellosis is an emerging disease of dogs in NSW with the principal risk being involvement in feral pig hunting. Veterinarians should consider this diagnosis in any dog that presents with reproductive tract signs, back pain or lameness, particularly if the dog has a history of feral pig exposure. The extent to which infected dogs present an ongoing risk to humans and/or other dogs in the household is unknown but plausible. Therefore all people in close contact with infected dogs such as hunters, household contacts and veterinary personnel should take precautions to prevent zoonotic transmission. More research into the natural history of infection and treatment is needed to formulate more evidenced-based advice on clinical management of infected dogs.
